# Exercise-Induced Changes in Hemodynamics, Hormones, Electrolytes, and Inflammatory Markers in Veteran Athletes with and without Coronary Atherosclerosis

**DOI:** 10.1249/MSS.0000000000003674

**Published:** 2025-02-17

**Authors:** SYLVAN L. J. E. JANSSEN, VINCENT L. AENGEVAEREN, FEMKE DE VRIES, GEERT KLEINNIBBELINK, ALMA M. A. MINGELS, MARIA T. E. HOPMAN, AREND MOSTERD, BIRGITTA K. VELTHUIS, NIELS P. RIKSEN, THIJS M. H. EIJSVOGELS

**Affiliations:** 1 Department of Medical BioSciences, Radboud University Medical Center, Nijmegen, THE NETHERLANDS; 2 Department of Cardiology, Radboud University Medical Center, Nijmegen, THE NETHERLANDS; 3 Central Diagnostic Laboratory, Maastricht University Medical Center, Maastricht, THE NETHERLANDS; 4 Department of Cardiology, Meander Medical Center, Amersfoort, THE NETHERLANDS; 5 Department of Radiology, University Medical Center Utrecht, Utrecht, THE NETHERLANDS; 6 Department of Internal Medicine, Radboud University Medical Center, Nijmegen, THE NETHERLANDS

**Keywords:** CORONARY ARTERY DISEASE, EXERCISE, PHYSIOLOGY, ATHLETES, IMMUNOLOGY

## Abstract

**Aims:**

Middle-aged and older male athletes have more coronary atherosclerosis than less active peers. We aimed to explore mechanisms that can contribute to this accelerated coronary atherosclerosis by comparing exercise-induced changes in hemodynamic factors, circulating hormones, electrolytes, and inflammatory markers across athletes with and without coronary atherosclerosis.

**Methods:**

Fifty-nine male athletes recruited from the MARC-2 study were stratified as controls (coronary artery calcium score [CACS] = 0, *n* = 20), high CACS (≥300 Agatston units or ≥75th Multi-Ethnic Study of Atherosclerosis percentile, *n* = 20), or significant stenosis (≥50% in any coronary artery, *n* = 19). At rest, during an exhaustive endurance cycling test and after 3 h of recovery, we measured blood pressure and blood concentrations of parathyroid hormone (PTH), calcium, magnesium, phosphate, C-reactive protein (CRP), IL-6, IL-1RA, IL-10, intercellular adhesion molecule 1 (ICAM-1), VCAM-1, and E-selectin.

**Results:**

Fifty-eight participants completed the exercise test (76 ± 14 min). All biomarkers changed during exercise, except CRP, ICAM-1, and VCAM-1. Systolic blood pressure, PTH, calcium, phosphate, IL-6, IL-1RA, and E-selectin concentrations increased during exercise. By contrast, diastolic blood pressure and magnesium concentrations decreased during exercise. The magnitude of exercise-induced responses of hemodynamic factors, circulating hormones, electrolytes, cytokines, and adhesion molecule concentrations did not, however, differ across groups.

**Conclusions:**

Blood pressure, hormone, electrolyte, and cytokine concentrations changed after an exhaustive endurance exercise test, but the magnitude of these responses did not differ between athletes with versus without coronary atherosclerosis. These findings suggest that accelerated coronary atherosclerosis in endurance athletes may not be explained by differences in responses to exercise but by differences in exercise exposure or other mechanisms not assessed in this study.

Exercise training is associated with improved cardiovascular risk factors and superior longevity (1–4). However, recent studies found a higher prevalence of coronary atherosclerosis among lifelong recreational athletes compared with sedentary peers (4–8), which was related to higher lifelong exercise volumes (4,9) and training at very vigorous exercise intensity (10,11). Interestingly, the most active athletes had fewer mixed and more often only calcified plaques, which suggests they had a less deleterious plaque composition (4). Furthermore, it was shown that a high cardiorespiratory fitness can partially attenuate the risk associated with high coronary artery calcification scores (12). These findings may explain the increased longevity seen in endurance athletes despite the increased presence of more coronary atherosclerosis in the most active athletes (4). Nevertheless, the underlying mechanisms responsible for the accelerated coronary atherosclerosis observed in middle-aged and older athletes are still under debate (13).

Several hypotheses have been proposed on how exercise may enhance coronary artery calcification (CAC) (14–16). Hypertension is a known risk factor for coronary atherosclerosis (17), and exercise is known to increase systolic blood pressure (SBP) in an intensity-dependent manner (18,19). As such, prolonged SBP elevations during (vigorous) exercise may affect the risk of CAC. Furthermore, an acute bout of exercise can affect hormone and electrolyte concentrations involved in vascular calcifications. Parathyroid hormone (PTH) concentrations increase after exercise (20,21), and higher PTH concentrations are associated with greater atherosclerotic burden (22–24). Moreover, increases in total calcium and phosphate concentrations have been reported after exercise (25). Patients with hyperparathyroidism and associated hypercalcemia have an increased risk of cardiovascular events (26). Similarly, higher phosphate levels have been associated with vascular calcifications even at phosphate concentrations within normal ranges (27). Hypomagnesemia may occur after exercise (28) and is also associated with more coronary atherosclerosis (29). More pronounced exercise-induced changes in these electrolytes may affect the risk of CAC. In addition, inflammation is known to play a central role in coronary atherosclerosis (30), and although regular physical activity lowers systemic inflammation (31), maximal exercise can produce a proinflammatory milieu (32). Interleukin 6 (IL-6), secreted by skeletal muscles during physical activity, and higher levels of adhesion molecules may recruit leukocytes to the vessel wall, promoting atheroma formation (31). Unfortunately, none of these hypotheses have been confirmed by observational data in athletes with versus without coronary atherosclerosis.

Therefore, we aimed to compare resting and exercise-induced changes in blood pressure (BP) and blood biomarkers potentially associated with coronary atherosclerosis between 1) athletes without coronary atherosclerosis, 2) athletes with high coronary artery calcium scores (CACS), and 3) athletes with a significant coronary stenosis. For this purpose, all participants performed a standardized strenuous endurance exercise test under controlled laboratory conditions with serial physiological measurements and blood sampling.

## METHODS

### Participants

Participants of the Measuring Athletes’ Risk of Cardiovascular events 2 (MARC-2, 2019–2020) study (10) were invited for this follow-up study (2020) and stratified by their degree of coronary atherosclerosis. We aimed to recruit three distinct groups: 1) controls—athletes with CACS = 0 Agatston units (AU) and without plaques on coronary computed tomography angiography; 2) high CACS—athletes with CACS ≥300 AU or CACS ≥75th Multi-Ethnic Study of Atherosclerosis (MESA) percentile (33) without significant stenosis; 3) significant stenosis—athletes with ≥50% stenosis in any coronary artery but cleared to perform exercise by a cardiologist, which was generally done after a stress imaging test without signs of ischemia (34). We examined male athletes only because 1) the higher prevalence of coronary atherosclerosis was found among male (4,7,8), but not female (35), lifelong athletes; 2) 90%–95% of exercise-related cardiac arrests occur in men (36); and 3) because the MARC cohort (10,37) consisted of men only. Participants were eligible for this study if they were able to perform a ±1.5 h exercise test on a stationary bike. Participants were excluded when they (i) had a stent in any coronary artery, (ii) had undergone coronary artery bypass surgery, or (iii) were not cleared for intense exercise training by a cardiologist after the MARC-2 coronary computed tomography scan findings. Beyond CACS and stenosis degree, we reported plaque characteristics previously collected and included plaque number and plaque types from the coronary computed tomography angiography. Habitual exercise characteristics were previously reported during MARC-2 measurements (10), and we used the average exercise dose from age 12 yr until participation in the MARC-2 study. We report years of exercise, average sessions per week, duration per session, hours of exercise per week, metabolic equivalent of task (MET)-hours per week, and the intensity at which exercise was performed (light <3 MET, moderate 3–6 MET, vigorous 6–9 MET, and very vigorous ≥9 MET) as a percentage of MET-hours per week. All participants provided written informed consent before participation. The Medical Research Ethical Committee region Arnhem-Nijmegen approved this study (NL74326.091.20), which was conducted in accordance with the Declaration of Helsinki.

### Procedures and Materials

#### Screening

Participants came to our research center for a single study visit (~6 h) between October 2020 and January 2021. They were instructed to refrain from exercise ≥36 h before the measurements, and all participants underwent a screening consisting of medical history taking and physical examination, including BP measurements and a resting electrocardiogram.

At baseline, BP was measured after at least 5 min of seated rest with the arm resting at heart level and no conversation with participants. At least three consecutive BP measurements were taken from both arms (two measurements on the left arm and at least one on the right) using an automated device (Omron M3 Comfort, Omron Healthcare, Japan). Mean resting BP in millimeters of mercury and mean resting heart rate (HR) in beats per minute (bpm) were calculated from these measurements. Height (cm) was measured with a wall-mounted measuring tape. Weight (kg) was measured using a calibrated digital scale (Seca 770, Seca Medizinische Messsysteme und Waagen, Germany).

#### Exercise test

The exercise test was performed on a stationary bike (Lode Excalibur Sport, Lode B.V. Medical Technology, Groningen, the Netherlands) in a room with standardized room temperature (18°C). The exercise protocol (Supplemental Fig. 1, Supplemental Digital Content, http://links.lww.com/MSS/D198) started after 5 min of seated rest (baseline). Participants performed a 30-min warm-up with a gradually increasing workload at a cadence of 80–100 revolutions per minute until they reached a stable HR at 70% of their maximum HR, which was obtained from (i) training data, (ii) a previously performed maximal exercise test, or (iii) an estimation based on age (208–0.7 × age) (38). When 70% of maximum HR was reached, the workload was kept stable until 30 min of warm-up had passed. After that, the workload increased every 3 min by 5% until volitional exhaustion, defining the end of the exercise test, followed by a cooldown of 3 min at 50 W. Lactate concentrations were assessed at baseline and 2 min after exercise cessation (Arkray Lactate Pro 2, Axonlab AG, Baden, Switzerland).

#### Hemodynamic parameters

HR (Quark C12x, COSMED srl, Rome, Italy & Polar V800; Polar, Kempele, Finland) was continuously monitored during the exercise test. The rating of perceived exertion was assessed using the Borg scale (39) at baseline, every 10 min during the exercise test, at maximal exertion and 2 min postexercise.

During exercise, BP was measured with an automatic cuff (SunTech Tango+, SunTech Medical Inc., Morrisville, NC) around the arm without an IV or (in case the automatic cuff failed due to excessive arm movement) a hand sphygmomanometer (Welch Allyn, Skaneateles Falls, NY). Maximum HR and wattage were determined at the end of the test. We assessed the prevalence of an exaggerated BP response, defined as a maximum SBP during exercise ≥210 mm Hg (40).

### Biomarkers

#### Blood sampling

Blood samples (±15 mL per withdrawal) were collected before exercise (baseline), at maximal exertion and 60 and 180 min postexercise. Samples were collected in serum-gel Vacutainer tubes (BD Vacutainer® SST™ II Advance 8.5 mL; Becton Dickinson, Franklin Lakes, NJ), lithium-heparin plasma tubes (plasma separator tubes, PST II, BD Vacutainer), and EDTA plasma tubes (K2EDTA tubes, BD Vacutainer). The serum tubes were allowed to clot for a minimum of 45 min. After centrifugation, serum and plasma were aliquoted, frozen (all done directly on-site), and stored at −80°C until analysis.

#### PTH, calcium, phosphate, and magnesium

PTH concentrations were analyzed on an IMMULITE 2000 analyzer (IMMULITE 2000 Intact PTH; Siemens Healthcare, Gwynedd, United Kingdom) with a reference interval of 1.2–7.1 pmol·L^−1^, according to the package insert. Total calcium concentrations were analyzed on a Cobas 6000 analyzer (Calcium Gen.2; Roche Diagnostics, Mannheim, Germany) with a limit of detection (LoD) of 0.20 mmol·L^−1^ and reference intervals of 2.15–2.50 mmol·L^−1^ for adults 18–60 yr old and 2.20–2.55 mmol·L^−1^ for adults 60–90 yr old, according to the package insert. Albumin concentrations were analyzed on a Cobas 6000 analyzer (Albumin BCP, Roche Diagnostics) with an LoD of 2 g·L^−1^ and reference intervals of 35–52 g·L^−1^, according to the package insert. As exercise hemoconcentration has a significant effect on calcium concentrations, the albumin-corrected calcium was calculated using the frequently used Payne equation (41): albumin-corrected [calcium] (mmol·L^−1^) = (0.02 × (40 – [albumin] (g·L^−1^))) + [total serum Ca] (mmol·L^−1^). Phosphate concentrations were measured on a Roche C8000 system, module C702 (PHOS2, Phosphate (Inorganic) ver.2, Roche Diagnostics) with an LoD of 0.10 mmol·L^−1^ and a reference interval of 0.81–1.45 mmol·L^−1^, according to the package insert. Magnesium concentrations were analyzed on a Cobas 6000 analyzer (Magnesium Gen.2, Roche Diagnostics) with an LoD of 0.10 mmol·L^−1^ and a reference interval of 0.66–1.07 mmol·L^−1^ for adults 18–60 yr old and 0.66–0.99 mmol·L^−1^ for adults 60–90 yr old, according to the package insert.

#### Cytokines and adhesion molecules

C-reactive protein (CRP) concentrations were measured using human CRP/CRP DuoSet ELISA kits (DY1707; R&D Systems, Minneapolis, MN). IL-6 concentrations were measured using Human IL-6 Quantikine HS ELISA kits (HS600C, R&D Systems). IL-10 and IL-1 receptor antagonist (IL-1RA) were using Human IL-10 Quantikine HS ELISA Kit (HS100C, R&D Systems) and Simple Plex Human IL-1RA/IL-1F3 Ella automated immunoassay system (SPCKC-PS-003151; ProteinSimple/Bio-Techne, Minneapolis, MN), respectively. Furthermore, we determined the concentrations of three cellular adhesion molecules. Soluble intercellular adhesion molecule 1 (ICAM-1) concentration was measured using Human ICAM-1/CD54 DuoSet ELISA kits (DY720, R&D Systems), soluble vascular cell adhesion molecule 1 (VCAM-1) using Human VCAM-1/CD106 DuoSet ELISA (DY809, R&D Systems), and E-selectin using Human E-selectin/CD62E DuoSet ELISA (DY724, R&D Systems). All assay kits are commercially available, and the concentrations were determined following the manufacturer’s instructions.

### Statistical Analysis

Data are presented as mean ± SD, median [interquartile range], or frequency (%). The normality of variables was tested using Shapiro–Wilk tests. The homogeneity of variance between groups was tested using Levene’s tests. One-way ANOVA tests were used to compare the means of normally distributed continuous variables. We used Kruskal–Wallis tests to compare means when continuous variables violated the ANOVA assumptions. Categorical variables were compared using Pearson chi-square tests or in case the expected count for any cell was below 5, with Fisher’s exact tests. If significant differences were found, pairwise comparisons with a Bonferroni correction for multiple testing were performed.

To test whether time-dependent changes in BP, PTH, electrolyte, cytokine, and adhesion molecule concentrations differed between groups, we performed a mixed model analysis using random intercepts. PTH, electrolyte, cytokine, and adhesion molecule concentrations were significantly skewed and therefore logarithmically transformed prior to the mixed model analyses. Time was described as a categorical variable for baseline, maximal exertion, and at 180 min postexercise. The interaction effect (group–time) between changes over time was subsequently tested. All statistical tests were performed using IBM SPSS Statistics 29 (IBM Corp., Armonk, New York), and *P* values less than 0.05 were considered statistically significant.

## RESULTS

### Participant characteristics

In total, 59 participants were recruited: *n* = 20 controls, *n* = 20 with high CACS, and *n* = 19 with significant coronary stenoses. One participant from the significant stenosis group did not complete the exercise test due to a knee injury and was therefore excluded from further analyses. Participant characteristics such as age (61 [58–69] yr), height, body mass index, and BP did not differ across study groups (Table 1). The high CACS group had a calcium score of 323 [195–610] AU, corresponding to the 84th [80–92] age-related MESA percentile, whereas the significant stenosis group had a calcium score of 681 [64–1312] AU, also corresponding to the 84th [72–94] age-related MESA percentile. In the significant stenosis group, a 50%–69% stenosis was more common (*n* = 12) than a ≥70% stenosis (*n* = 6).

**TABLE 1:** Baseline characteristics, total and split in different study groups.

Participant Characteristics	Total Cohort (*n* = 58)	Controls (*n* = 20)	High CACS (*n* = 20)	≥50% Stenosis (*n* = 18)	*P* Value
Age, yr	61 [58–69]	60 [57–69]	60 [58–67]	65 [61–69]	0.24
Height, cm	183 ± 7	182 ± 6	184 ± 6	182 ± 8	0.51
Body mass, kg	84.9 ± 10.2	81.0 ± 8.0	89.0 ± 10.0*^a^*	84.6 ± 11.2	0.04*
BMI, kg·m^−2^	25.3 ± 2.5	24.4 ± 2.0	26.2 ± 2.7	25.4 ± 2.5	0.08
BP					
Systolic, mm Hg	144 ± 14	141 ± 12	145 ± 13	146 ± 16	0.39
Diastolic, mm Hg	88 ± 10	86 ± 8	89 ± 12	88 ± 11	0.67
Resting HR, bpm	59 [55–70]	58 [52–64]	63 [55–71]	60 [55–72]	0.38
Expected maximum HR, bpm	166 ± 10	168 ± 7	166 ± 10	163 ± 12	0.36
Cardiovascular risk factors	27 (46.6)	0 (0.0)	13 (65.0)*^a^*	14 (77.8)*^a^*	<0.001*
Hypertension, *n* (%)	5 (8.6)	0 (0.0)	2 (10.0)	3 (16.7)	0.18
Antihypertensive drug(s), *n* (%)	3 (6.5)	0 (0)	1 (5)	2 (11.1)	0.78
Hypercholesterolemia, *n* (%)	23 (39.7)	0 (0)	11 (55.0)*^a^*	12 (66.7)*^a^*	<0.001*
Statin users, *n* (%)	24 (41.4)	0 (0)	11 (55.0)*^a^*	13 (72.2)*^a^*	<0.001*
Diabetes mellitus, *n* (%)	1 (1.7)	0 (0)	1 (5.0)	0 (0.0)	1.00
Family history of CV disease, *n* (%)	9 (15.5)	2 (10.0)	3 (15.0)	4 (22.2)	0.60
Smoking					0.43
No, *n* (%)	53 (91.4)	19 (95.0)	19 (95.0)	15 (83.3)	
Current or quit <2 yr ago, *n* (%)	5 (8.6)	1 (5.0)	1 (5.0)	3 (16.7)	
Plaque characteristics					
CACS, AU	185 [0–648]	0 [0–0]	323 [195–610]*^a^*	681 [64–1312]*^a^*	<0.001*
CACS ≥ 300 AU, *n* (%)	21 (36.2)	0 (0.0)	10 (50.0)*^a^*	11 (61.1)*^a^*	<0.001*
MESA percentile	79 [0–88]	0 [0–0]	84 [80–92]*^a^*	84 [72–94]*^a^*	<0.001*
≥75th MESA percentile, *n* (%)	33 (56.9)	0 (0.0)	19 (95.0)*^a^*	14 (77.8)*^a^*	<0.001*
Significant (≥50%) stenosis, *n* (%)	18 (31.0)	0 (0.0)	0 (0.0)	18 (100.0)*^a,b^*	<0.001*
Stenosis 50%–69%, *n* (%)	12 (20.7)	0 (0.0)	0 (0.0)	12 (66.7)*^a,b^*	<0.001*
Stenosis ≥70%, *n* (%)	6 (10.3)	0 (0.0)	0 (0.0)	6 (33.3)*^a,b^*	<0.001*
Total number of plaques, *n*	11 [7–16]	0 [0–0]	11 [7–16]	11 [7–16]*^b^*	<0.001*
Calcified plaques*^b^*					
Presence, *n* (%)	35 (60.3)	–	19 (95.0)*^a^*	16 (88.9)*^a,b^*	<0.001*
Number, *n*	5 [3–9]	–	5 [3–9]	7 [2–10]*^b^*	<0.001*
Percentage of total plaques, %	50.0 [29.4–66.7]	–	51.7 [28.8–73.4]	48.5 [33.1–63.5]	0.60
Partially calcified plaques					
Presence, *n* (%)	33 (56.9)	–	18 (90.0)*^a^*	15 (83.3)*^a,b^*	<0.001*
Number, *n*	3 [2–7]	–	3 [2–8]	4 [2–6]*^b^*	<0.001*
Percentage of total plaques, %	37.5 [21.7–50.0]	–	38.8 [25.0–63.5]	34.3 [18.6–47.1]	0.30
Noncalcified plaques					
Presence, *n* (%)	17 (29.3)	–	8 (40.0)*^a^*	9 (50.0)*^a,b^*	<0.001*
Number, *n*	0 [0–1]	–	0 [0–1]	1 [0–2]*^b^*	0.002*
Percentage of total plaques, %	0.0 [0.0–14.3]	–	0.0 [0.0–7.0]	5.3 [0.0–16.5]	0.21
Lifelong exercise history*^c^*					
Years of exercise	43 [34–49]	40 [23–44]	44 [37–49]	46 [36–51]	0.07
Sessions per week, *n*	1.9 [1.2–2.7]	1.7 [1.1–2.5]	2.4 [1.2–2.8]	1.8 [1.2–2.7]	0.51
Duration per session, *h*	1.5 [1.3–1.9]	1.6 [1.4–1.8]	1.6 [1.3–2.0]	1.4 [1.3–1.9]	0.88
Exercise duration per week, *h*	2.8 [1.8–4.7]	2.9 [1.6–4.2]	2.8 [2.2–4.9]	2.9 [1.6–4.3]	0.63
MET-minutes per week	1477 [861–2188]	1510 [871–2160]	1350 [916–2211]	1411 [793–2202]	0.87
% of exercise time performed					
Light intensity, %	0.0 [0.0–0.0]	0.0 [0.0–0.0]	0.0 [0.0–0.0]	0.0 [0.0–0.0]	1.00
Moderate intensity, %	6.1 [0.0–19.4]	1.6 [0.0–9.3]	18.2 [2.0–38.6]*^a^*	6.3 [0.5–14.7]	0.011*
Vigorous intensity, %	57.1 [15.5–84.1]	67.8 [25.3–83.3]	53.6 [8.7–72.6]	57.1 [6.9–93.0]	0.56
Very vigorous intensity, %	25.6 [0.0–66.5]	24.9 [14.8–69.4]	22.6 [0.0–51.4]	30.3 [0.0–71.3]	0.67

Statistics could not be computed because stenosis degrees (“50%–69%” versus “≥70%”) were mutually exclusive.

* *P* < 0.05.

*^a^* Pairwise comparison, significantly different from controls.

*^b^* Pairwise comparison, significantly different from the High CACS group.

*^c^* Lifelong exercise history was since the age of 12 yr up to MARC-2 participation in 2019–2020.

BMI, body mass index; AU, Agatston units; CV, cardiovascular.

– Statistics could not be computed because this condition was prevalent in all participants.

### Exercise characteristics and hemodynamic parameters

**FIGURE 1:**
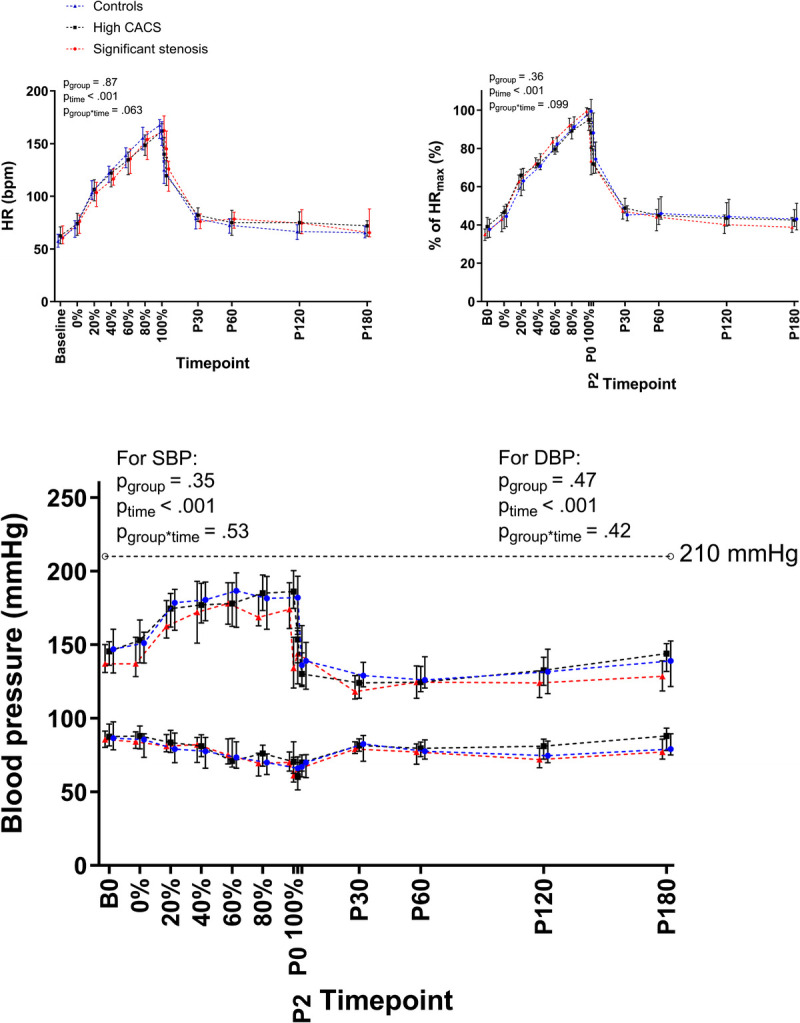
Time-dependent changes in HR, percentage of maximum HR (% of HR_max_), and BP for the control group (in *blue*), the high CACS group (in *black*), and the significant stenosis group (in *red*). HR and percentage of maximum HR gradually increased during the exercise test (*P*_time_ < 0.001) but did not differ across groups (*P*_group_ = 0.87 and *P*_group_ = 0.36, respectively). SBP increased during exercise but was significantly lower postexercise than at baseline (*P*_time_ < 0.001). Diastolic BP decreased during exercise and returned to baseline levels during recovery (*P*_time_ < 0.001). Data are presented at baseline, at 0%, 20%, 40%, 60%, 80%, and 100% completion of the exercise test, and at *t* = 0, *t* = 2, *t* = 30, *t* = 60, *t* = 120, and *t* = 180 min after exercise cessation (P0, P2, P30, P60, P120, P180, respectively).

Participants exercised for 76 ± 14 min and obtained a peak HR of 164 ± 13 bpm (98% [95%–102%] of maximum HR) and lactate concentration of 8.5 [6.6–13.1] mmol·L^−1^, which did not differ across groups (Table 2). Workload gradually increased over time up to a peak value of 209 ± 38 W (*P*_time_ < 0.001) and did not differ across groups (*P*_group_ = 0.43). HR increased from 59 [55–70] bpm at baseline to 164 ± 13 bpm at peak exercise (*P*_time_ < 0.001) and did not differ across groups (*P*_group_ = 0.87, Figure 1). Rating of perceived exertion gradually increased up to 19 [19,20] at peak exercise (*P*_time_ < 0.001) and did not differ across groups (*P*_group_ = 0.38), nor did the change over time differ across groups (*P*_group–time_ = 0.74). SBP was 144 ± 14 mm Hg at baseline and increased during exercise to a peak SBP of 192 ± 21 mm Hg (*P*_time_ < 0.001). Ten participants (17.2%) showed an exaggerated BP response (i.e., maximum SBP during exercise ≥210 mm Hg), but the prevalence did not differ across groups (*P* = 0.83). During recovery, SBP dropped to values significantly lower than at baseline with lowest SBP values of 125 ± 12 at 60 min postexercise (*P*_time_ < 0.001). DBP was 88 ± 10 mm Hg at baseline, decreased to 63 ± 12 mm Hg at peak exercise, and gradually recovered postexercise (*P*_time_ < 0.001). Resting and exercise-induced changes in SBP and diastolic blood pressure (DBP) did not differ across groups (*P*_group–time_ = 0.53 and 0.42, respectively).

**TABLE 2:** Exercise test performance, total and split for different study groups.

Exercise Test Performance	Total Cohort (*n* = 58)	Controls (*n* = 20)	High CACS (*n* = 20)	≥50% Stenosis (*n* = 18)	*P* Value
Exercise duration, min	76 ± 14	77 ± 12	77 ± 17	75 ± 12	0.88
Peak HR, bpm	164 ± 13	166 ± 12	160 ± 13	162 ± 17	0.43
Exercise intensity, %HR_max_	97.7 [94.8–101.8]	99.1 [96.6–102.2]	95.7 [93.1–100.6]	99.1 [94.2–105.3]	0.20
Maximum workload, W	209 ± 38	215 ± 33	213 ± 43	198 ± 36	0.34
Maximum workload, W·kg^−1^	2.48 ± 0.48	2.65 ± 0.37	2.41 ± 0.53	2.37 ± 0.49	0.13
Maximum SBP, mm Hg	192 ± 21	187 ± 18	195 ± 19	195 ± 27	0.51
Exaggerated BP response	10 (17.2)	3 (15.0)	3 (15.0)	4 (21.1)	0.83
Lactate 2 min postexercise, mmol·L^−1^	8.5 [6.6–13.1]	10.9 [6.9–14.0]	8.2 [5.8–11.9]	8.3 [6.8–13.3]	0.58
Maximum RPE	19 [19–20]	20 [19–20]	19 [19–20]	19 [18–20]	0.30

%HR_max_, percentage of expected maximum HR; RPE, rating of perceived exertion scale (6 = very, very light; 20 = maximum exertion); maximum workload (W·kg^−1^), maximum workload in watts per kilogram of body weight.

### PTH, calcium, phosphate, and magnesium concentrations

**FIGURE 2:**
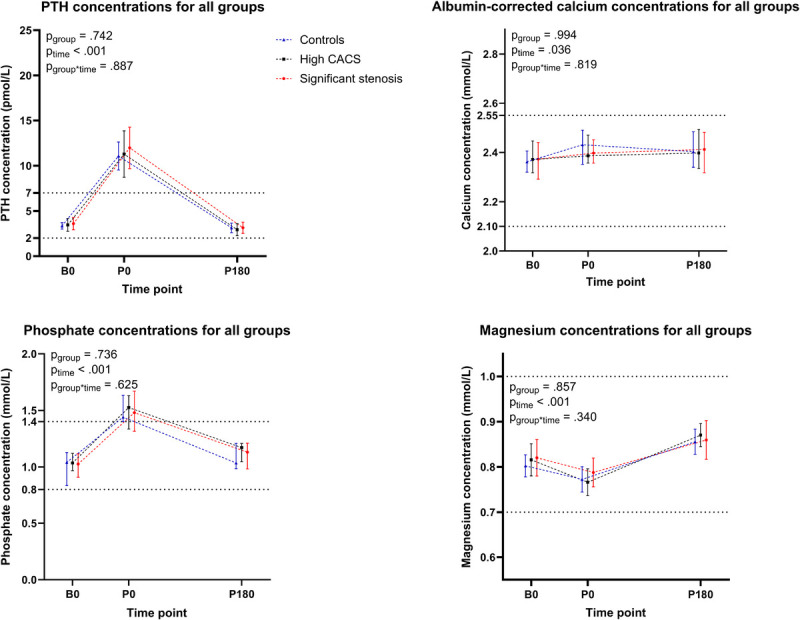
Time-dependent changes in PTH, calcium, phosphate, and magnesium concentrations at baseline, at maximal exertion and 180 min postexercise for the control group (in *blue*), the high CACS group (in *black*), and the significant stenosis group (in *red*). PTH concentrations increased during exercise (*P*_time_ < 0.001) but did not differ across groups (*P*_group_ = 0.742). Albumin-corrected calcium concentrations showed a slight increase (*P*_time_ = 0.036) but did not differ across groups (*P*_group_ = 0.994). Phosphate concentrations increased during exercise (*P*_time_ < 0.001) but did not differ across groups (*P*_group_ = 0.736). Magnesium concentrations decreased during exercise (*P*_time_ < 0.001) but did not differ across groups (*P*_group_ = 0.857). Data are presented at baseline (B0), at maximal exertion (P0), and 180 min postexercise (P180).

The PTH concentration was 3.4 [2.6–4.1] pmol·L^−1^ at baseline, increased to 11.4 [7.7–14.7] pmol·L^−1^ at peak exercise, and then decreased to 2.8 [2.1–3.8] pmol·L^−1^ after 180 min of recovery (*P*_time_ < 0.001, Figure 2), but no differences across groups were observed (*P*_group_ = 0.74). Albumin-corrected calcium concentrations demonstrated a slight increase from 2.37 [2.31–2.44] mmol·L^−1^ at baseline to 2.40 [2.36–2.48] mmol·L^−1^ at peak exercise and remained at 2.40 [2.33–2.48] mmol·L^−1^ after 180 min of recovery (*P*_time_ = 0.036). The albumin-corrected calcium concentrations did not differ across groups (*P*_group_ = 0.994). Phosphate concentrations increased from 1.0 [0.9–1.1] mmol·L^−1^ at baseline to 1.5 [1.3–1.6] mmol·L^−1^ at peak exercise and normalized again to 1.1 [1.0–1.2] mmol·L^−1^ during recovery (*P*_time_ < 0.001) but demonstrated no differences across groups (*P*_group_ = 0.74). Magnesium concentrations were 0.82 [0.78–0.87] mmol·L^−1^ at baseline, decreased to 0.78 [0.74–0.82] mmol·L^−1^ at peak exercise, and then increased to 0.85 [0.82–0.92] mmol·L^−1^ after recovery (*P*_time_ < 0.001), but no differences between groups were observed (*P*_group_ = 0.86). The individual participant-level data for PTH, albumin-corrected calcium, phosphate, and magnesium concentrations can be found in Supplemental Figures 2–5 (Supplemental Digital Content, http://links.lww.com/MSS/D198).

### Cytokine concentrations

**FIGURE 3:**
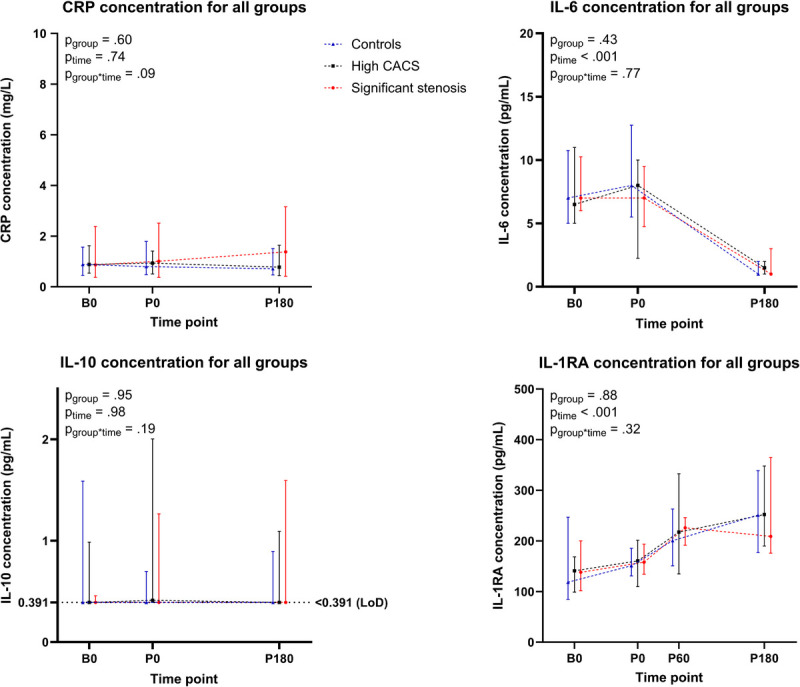
Time-dependent changes in cytokine concentrations at baseline, at maximal exertion and 180 min postexercise for the control group (in *blue*), the high CACS group (in *black*), and the significant stenosis group (in *red*). CRP concentrations did not change during or after exercise (*P*_time=_0.741) and did not differ across groups (*P*_group_ = 0.604). IL-6 concentrations were slightly increased at maximal exertion and decreased after exercise (*P*_time_ < 0.001) but did not differ across groups (*P*_group_ = 0.875). IL-10 concentrations mostly were below the limit of detection (LoD) and did not differ across groups (*P*_group_ = 0.798) nor over time (*P*_time_ = 0.965). IL-1RA concentrations increased during exercise (*P*_time_ < 0.001) but did not differ across groups (*P*_group_ = 0.881). Data are presented at baseline (B0), at maximal exertion (P0), and at 60 and 180 min postexercise (P60 and P180, respectively).

The CRP concentration was 0.9 [0.4–1.7] mg·L^−1^ at baseline and did not significantly change over time (*P*_time_ = 0.74) or across groups (*P*_group_ = 0.60, Figure 3). IL-6 increased from 7.0 [5.2–11.3] pg·mL^−1^ at baseline to 8.4 [4.3–11.1] pg·mL^−1^ at peak exercise and decreased to 1.3 [1.0–1.9] pg·mL^−1^ after 180 min of recovery (*P*_time_ < 0.001), but responses did not differ across groups (*P*_group_ = 0.43). For IL-10, *n* = 107 samples (61.8%) were below the LoD of 0.391 pg·mL^−1^ and could thus not be quantified. Consequently, the IL-10 concentrations were excluded from further statistical analyses. IL-1RA increased from 122 [99–186] pg·mL^−1^ at baseline to 154 [128–189] pg·mL^−1^ at peak exercise, 223 [162–263] pg·mL^−1^ after 60 min of recovery, and even 245 [184–342] pg·mL^−1^ after 180 min of recovery (*P*_time_ < 0.001). No group differences in IL-1RA concentrations were observed (*P*_group_ = 0.88). The individual participant-level data for CRP, IL-6, IL-1RA, and IL-10 can be found in Supplemental Figures 6–9 (Supplemental Digital Content, http://links.lww.com/MSS/D198).

### Adhesion molecule concentrations

**FIGURE 4:**
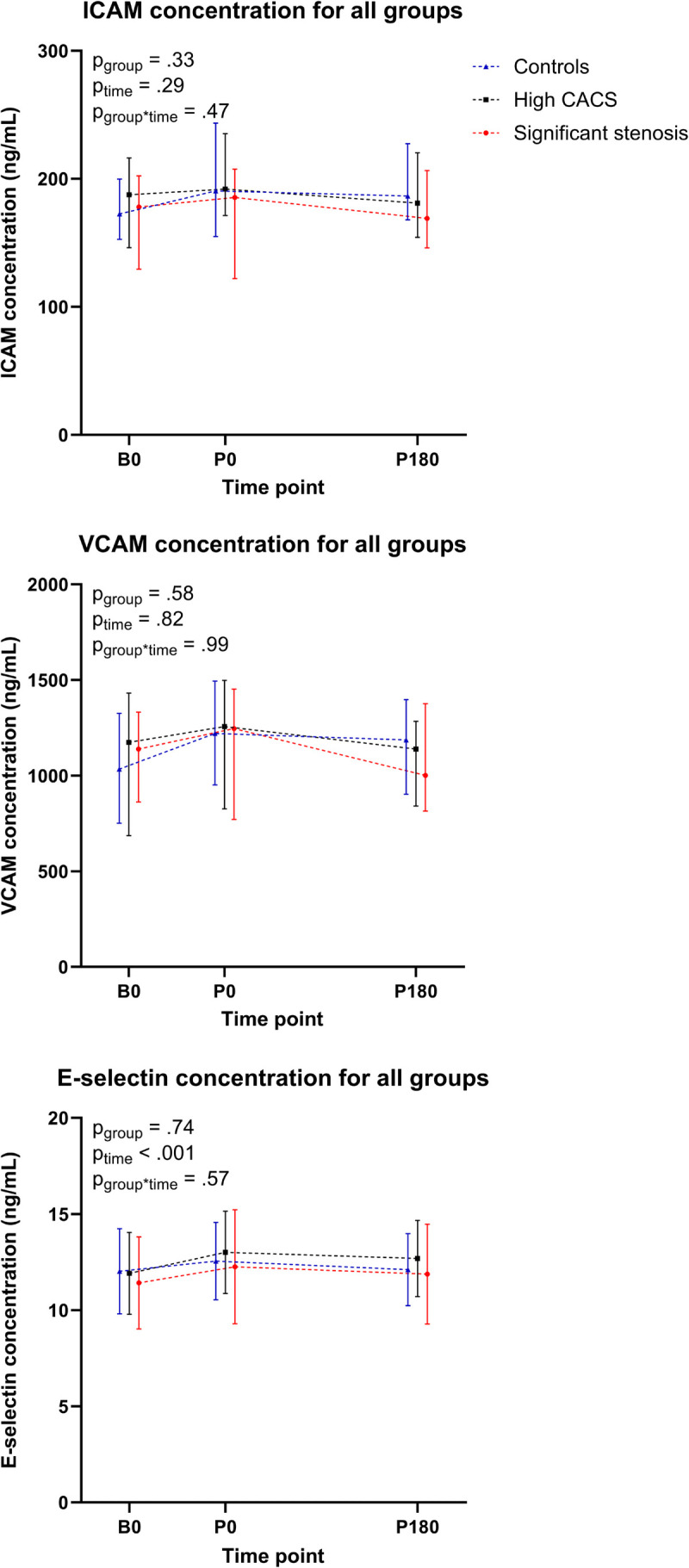
Time-dependent changes in ICAM-1, VCAM-1, and E-selectin concentrations at baseline, at maximal exertion and 180 min postexercise for the control group (in *blue*), the high CACS group (in *black*), and the significant stenosis group (in *red*). ICAM-1 concentrations did not change during or after exercise (*P*_time=_0.284) and did not differ across groups (*P*_group_ = 0.804). VCAM-1 concentrations did not change during or after exercise (*P*_time_ = 0.840) and did not differ across groups (*P*_group_ = 0.332). E-selectin concentrations increased a little during exercise (*P*_time_ < 0.001) and returned to baseline after 180 min recovery but did not differ across groups (*P*_group_ = 0.523). Data are presented at baseline (B0), at maximal exertion (P0), and 180 min postexercise (P180).

ICAM-1 concentrations were 184 [155–207] ng·mL^−1^ at baseline and did not change during exercise (191 [161–234] ng·mL^−1^ at peak exercise) or recovery (183 [155–221] ng·mL^−1^, *P*_time_ = 0.29), with similar responses across groups (*P*_group_ = 0.33, Figure 4). VCAM-1 concentrations were 1160 [887–1417] ng·mL^−1^ at baseline and did not change during exercise (1204 [829–1482] ng·mL^−1^ at peak exercise) or recovery (1145 [861–1321] ng·mL^−1^, *P*_time_ = 0.82), and these responses were similar across groups (*P*_group_ = 0.58). E-selectin concentrations significantly increased from 11.7 [7.8–14.0] ng·mL^−1^ at baseline to 12.4 [8.4–15.7] ng·mL^−1^ at peak exercise and returned to 11.2 [8.6–15.0] ng·mL^−1^ after 180 min of recovery (*P*_time_ < 0.001). However, the magnitude of E-selectin increases did not differ across groups (*P*_group_ = 0.74). The individual participant-level data for ICAM-1, VCAM-1, and E-selectin concentrations can be found in Supplemental Figures 10–12 (Supplemental Digital Content, http://links.lww.com/MSS/D198).

## DISCUSSION

We investigated potential mechanisms that can contribute to the higher prevalence of coronary atherosclerosis in middle-aged and older male athletes using a lab-based controlled endurance cycling test with repeated blood sampling. We observed time-dependent changes in BP, hormone, electrolyte, cytokine, and adhesion molecule concentrations. However, the magnitude of these exercise-induced responses was similar across groups. These findings suggest that accelerated atherosclerosis in recreational endurance athletes may be caused by differences in exercise exposure (i.e., volume or intensity differences) rather than interindividual differences in responses to a controlled exercise bout. In addition, other mechanisms that were not assessed in our study may also contribute to the observed accelerated atherosclerosis in veteran athletes.

### Blood pressure

In line with our hypothesis, SBP increased with an increasing workload while DBP decreased (18,42). With an average maximum of 192 ± 21 mm Hg at peak exercise and 10 participants demonstrating an exaggerated BP response (i.e., maximum SBP during exercise ≥210 mm Hg), our exercise protocol was appropriate to assess exercise-induced BP responses. The BP responses to this isolated and standardized exercise bout did not reveal differences across groups. However, the exposure to exercise-induced BP elevations during real-world endurance exercise (many hours per week at high intensity) may differ and thereby still contribute to accelerated coronary atherosclerosis in athletes because hypertension is a known major risk factor for CAC progression (43).

### PTH and electrolytes

High PTH concentrations are associated with aortic medial calcification (44) and the risk of atherosclerotic disease (21). The observed acute increase in PTH concentrations during exercise and the subsequent decrease after recovery align with previous studies (25,45,46), demonstrating that exercise of sufficient intensity and duration modifies calcium homeostasis and calciotropic hormone levels. Interestingly, Maïmoun et al. (46) only found a PTH increase after high-intensity exercise (at +15% above the ventilatory threshold), with no increase after lower-intensity exercise, which suggests that exercise intensity is important for PTH release. This aligns with the observation that exercise intensity but not volume was associated with the progression of coronary atherosclerosis during the 6-yr follow-up in the MARC-2 study (10). Therefore, athletes who train multiple hours per week, often at high intensity, may expose themselves repeatedly to PTH elevations, which could potentially contribute to accelerated coronary atherosclerosis. This indicates that our exercise protocol was of sufficient duration and intensity to increase PTH levels but did not reveal differences between the coronary atherosclerosis groups.

The exercise-induced increases in albumin-corrected calcium concentrations were statistically significant but clinically probably less relevant given the small absolute increase (2.37 [2.31–2.44] mmol·L^−1^ to 2.40 [2.36–2.48] mmol·L^−1^). Our findings align with some (45,47) but not all (48–50) previous studies, which may be due to heterogeneity in exercise duration, intensity, and type, as well as differences in participant characteristics such as training status and physical fitness (25) and timing of blood sampling. Another critical factor for the discrepancies is that some studies report ionized calcium, whereas others report total calcium, and not all studies adjust for exercise-induced hemoconcentration due to dehydration (25). Although calcium homeostasis theoretically could play a key role, the changes in calcium levels we observed were minor, making it less likely that exercise-induced increases in calcium concentrations play an essential role in the development of atherosclerosis in athletes.

Phosphate concentrations increased during exercise and normalized during recovery, which aligns with a previous study (51). The observed rise in serum phosphate is interesting because there is emerging evidence that high phosphate levels may promote vascular calcifications (27). In the MARC-2 cross-sectional analyses, higher resting phosphate concentrations were significantly associated with higher CAC scores (52). This suggests that repeated exposure to hyperphosphatemia after strenuous endurance exercise may also contribute to accelerated coronary atherosclerosis in lifelong athletes.

Magnesium concentrations decreased during exercise, probably via excessive sweat loss and urinary excretion (53) and a shift to adipocytes and skeletal muscles (54,55). Lower serum magnesium levels are associated with coronary artery disease (56) and CAC (57,58). Interestingly, the effect of low serum magnesium concentrations on CAC risk was even more significant among patients with higher serum phosphate concentrations (59). As we observed both higher phosphate and lower magnesium levels postexercise, these findings suggest that exercise-induced electrolyte changes may play a role in accelerated coronary artery calcification in highly active athletes.

### Cytokine concentrations

We assessed circulating concentrations of pro- and anti-inflammatory cytokines and adhesion molecules. IL-6 is a proinflammatory cytokine strongly associated with CVD risk (60). We observed that, during exercise, IL-6 increased, which aligns with literature demonstrating (large) increases immediately after strenuous exercise (61,62). The magnitude of inflammatory responses depends on the intensity and duration of exercise (61), confirming that our exercise protocol was appropriate to elicit exercise-induced changes in concentration of circulating cytokines. Interestingly, IL-6 decreased below baseline values during recovery, which was also reported before (63). These findings demonstrate that exercise, depending on the timing of blood sampling, can both increase and decrease IL-6 concentrations.

Immediately after exercise, the increase in proinflammatory IL-6 concentrations may contribute to atheroma formation. During recovery, however, the lower IL-6 concentrations may protect against atherosclerosis. Thus, on the one hand, higher IL-6 and adhesion molecule concentrations may promote atheroma formation by recruiting leukocytes to the vessel wall (31). At the same time, IL-6 triggers the release of IL-1RA and IL-10, which have anti-inflammatory properties and may protect against atherosclerosis (31). The role of IL-6 in accelerated atherosclerosis in athletes, therefore, warrants further investigation.

IL-1RA is an endogenous antagonist of the IL-1 receptor, produced mainly by myeloid cells in response to cytokines, including IL-6. We observed that IL-1RA increased during exercise and kept increasing up to 180 min postexercise, demonstrating the highest concentrations at 60 and 180 min postexercise, which aligns with previous findings (61,63). The magnitude of the IL-1RA concentrations aligns with values previously found in physically active males (64).

The CRP concentrations did not change during exercise or within 180 min postexercise, which aligns with previous findings demonstrating that CRP release peaks around 24 to 48 h postexercise (65). Summarizing the cytokine responses, we found an increase in IL-6 immediately postexercise and a subsequent increase in the “protective” anti-inflammatory cytokine IL-1RA during recovery, accompanied by a rapid decline in IL-6 concentrations. This demonstrates that exercise can have both pro- and anti-inflammatory responses, and more research is needed to unravel the exact effects of multiple types, intensities, and durations of exercise on the immune system.

### Adhesion molecule concentrations

Elevated concentrations of cell adhesion molecules (CAM) indicate inflammation and play a central role in the transmigration of monocytes to the vessel wall (66). Elevated CAM might, thus, promote vascular inflammation and atheroma formation (66). Interestingly, high-intensity exercise may increase CAM concentrations in specific circumstances, possibly due to inflammatory activity, endothelial activation, by shedding the CAM from the endothelial cell surface (due to elevated shear stress) (66) or via adrenergic mechanisms (67). In our participants, however, ICAM-1 and VCAM-1 concentrations demonstrated no changes over time. This aligns with several previous studies, which found no changes in soluble ICAM-1 and VCAM-1 concentrations after high-intensity exercise in healthy, trained men [3] or healthy controls (68,69). The absence of exercise-induced ICAM and VCAM elevations after our strenuous exercise test suggests that these adhesion molecules most likely play no or only a minor role in accelerated coronary atherosclerosis in athletes.

E-selectin is a recognized marker of endothelial activation (70), which plays a central role in the transmigration of monocytes (66). The observed increase after exercise aligns with a previous study in sickle cell trait carriers and healthy controls (69). Nonetheless, the observed increase was relatively subtle and heterogeneous among individuals with no differences across groups, suggesting that exercise-induced E-selectin elevations likely play no significant role in atherosclerosis in athletes.

### Strengths and limitations

To our knowledge, this is the first controlled study in a lab setting that deeply phenotypes responses to exercise in male recreational athletes with different degrees of coronary atherosclerosis. We serially measured multiple outcomes and used high-sensitivity ELISA and Ella techniques to quantify cytokine concentrations. This enables us to investigate the potential underlying mechanisms for accelerated coronary atherosclerosis in the population of concern: highly active male recreational athletes (35). Nevertheless, our study has several limitations. First, we used a cross-sectional observational study design to investigate whether exercise-induced responses to high-intensity endurance exercise differed between athletes with different degrees of coronary atherosclerosis, hoping to find clues that certain factors are associated with coronary atherosclerosis. However, atherosclerosis is a lifelong effect, and the cross-sectional nature is not suitable for studying the causality of these factors. Nevertheless, studying associations can be an important first step to gain more insight into atherosclerosis development in athletes. Second, participants performed a standardized yet personalized exercise test. Consequently, the absolute workload may differ between individuals. Nevertheless, the exercise intensity and duration did not differ across groups, nor did the measures for relative intensity (i.e., HR and percentage of maximum HR). Third, we only studied a selection of possible underlying mechanisms for CAC. We did not investigate the potential roles of genetics, psychological stress, dietary intake, exercise-induced mechanical stress on the vessel wall, or disrupted laminar flow in the coronary arteries. These have also been proposed as possibly related mechanisms in coronary atherosclerosis (11), and their possible contribution in CAC progression remains to be determined in future studies. Fourth, a nonactive control group was lacking as we aimed to study the exercise-induced responses in athletes with different degrees of coronary atherosclerosis. Therefore, we cannot translate our findings to a less active population, and we cannot say which markers may respond differently between sedentary individuals and athletes.

## CONCLUSIONS

BP, hormone, electrolyte, and cytokine concentrations changed significantly during or after an exhaustive endurance exercise test, but the magnitude of these responses did not differ between athletes with versus without coronary atherosclerosis. These findings suggest that accelerated coronary atherosclerosis in recreational endurance athletes may not be explained by interindividual differences in responses to exercise but by differences in exercise exposure or other mechanisms not assessed in this study.
